# Durable, Photostable Omniphobic Synthetic Leather Surfaces with Anti-Biofouling Properties for Hygienic Applications

**DOI:** 10.3390/polym16141983

**Published:** 2024-07-11

**Authors:** Hanna Lee, Jun Kyun Oh

**Affiliations:** Department of Polymer Science and Engineering, Dankook University, Yongin-si 16890, Republic of Korea

**Keywords:** synthetic leather, omniphobic, anti-biofouling, self-cleaning, hygiene

## Abstract

Globally, the public health domain is increasingly emphasizing the need for surfaces that can resist bacterial contamination, as the consumption of bacteria-infected substance may cause illnesses. Thus, this study aimed to modify polyurethane (PU) synthetic leather surfaces by coating their upper layer with fluorine-functionalized nano-silica particles (FNPs). This simple modification imparted omniphobic characteristics, realizing anti-biofouling and self-cleaning properties. The effectiveness in preventing bacterial adhesion was confirmed by the dip-inoculation method using *Escherichia coli* O157:H7 and *Staphylococcus epidermidis*. Bacterial adhesion was evaluated based on bacterial counts using the pour plate method and by directly enumerating from scanning electron microscopy images. The attachment of bacteria to the modified omniphobic FNPs-coated PU leather surface decreased by over 98.2% compared to that on the bare surface. We expect that the method developed in this study will significantly reduce or even eliminate the potential risks associated with various biological cross-contamination scenarios, thereby enhancing hygiene standards.

## 1. Introduction

Leather, which has been used since ancient times, holds a significant position as a material. It possesses advantages such as high tensile strength, high flexibility, high tear resistance, high resistance to abrasion, and excellent barrier properties against water and air [1]. Consequently, leather has been widely used in various industries related to clothing fabrics, furniture materials, automotive interiors, medical supplies, and so on. However, with the increasing interest in sustainable and environmentally friendly vegan leather, triggered from environmental and ethical considerations, as well as factors such as high cost, high weight, and complex dyeing and processing of natural leather, the demand for synthetic leather is gradually increasing. According to Consumer Reports in the USA, the projected growth of the global synthetic leather market is USD 36.0 billion by 2024, and the market is expected to maintain a compound annual growth rate of 7.8% until 2030, ultimately expanding to a size of USD 56.4 billion [2]. Among the various types of synthetic leather, polyurethane (PU)-based synthetic leather stands out for its texture and feel, which are similar to those of natural leather because its urethane structure resembles the peptide chain structure of collagen [3]. In many cases where synthetic leather is used, there is a high risk of external contaminants being transferred through body contact or hands, and the residual substances and other organic materials remaining on the surface provide nutrients to harmful microorganisms (e.g., *Escherichia coli*, *Pseudomonas aeruginosa*, *Staphylococcus*, and *Bacillus* bacteria), thus promoting their growth [4]. In the context of the recent trend of infectious diseases, this ultimately results in the spreading of diseases due to cross-contamination and transmission. Hence, there is a burgeoning interest in integrating anti-biofouling and self-cleaning functionalities into material surfaces to combat harmful factors. This underscores the pressing need for the development of efficient surface treatment technologies.

The anti-biofouling function is closely related to the wetting properties of surfaces. It is commonly observed at interfaces and associated with the adhesion behavior of contaminants [5]. When a liquid contacts the surface of a material, it either retains its droplet shape or spreads across the surface to create a thin layer. This can be confirmed by measuring the static contact angles (θ). Water contact angles below 10° indicate superhydrophilic nature; those from 10° to 90° indicate hydrophilic nature (in some cases, 10° to 65° specifically indicate hydrophilicity for biomatter); those from 90° to 150° indicate hydrophobic nature (in some cases, 65° to 150°); and those above 150° indicate superhydrophobic nature of the material [6]. Furthermore, a material that simultaneously repels polar molecules like water and non-polar molecules like oil is referred to as an omniphobic material [7]. Such a material exhibits a contact angle greater than 150°, indicating a significant repellent property against any liquid that comes into contact with it. Moreover, a surface with a sliding contact angle of less than 10° is recognized for its self-cleaning properties when a liquid is dropped on it, as the surface contaminants are easily removed along with the droplet [8]. Surfaces that show exceptional repulsion to liquids, such as those with anti-biofouling or self-cleaning properties, have been accomplished by reducing the surface energy through physical surface treatments, like creating nano-scale roughness or hierarchical structures, as well as chemical surface treatments involving the introduction of methyl groups, fluorinated compounds, and so on [9]. These modifications enable surfaces to repel both water and oil, regardless of the surface tension of the contaminants.

Currently, researchers are focusing on developing surface treatments that enable superhydrophilic and superhydrophobic modifications. These treatments aim to reduce cross-contamination risks and prevent the spread of pathogens such as bacteria, fungi, and algae on various surfaces [10]. In the context of superhydrophilic surface treatment, it refers to the generation of a surface characterized by a thin water adsorption layer formed through hydrogen bonds, enveloping nanorough surfaces [11]. The water layer prevents hydrophobic (non-polar) contaminants from adhering to the surfaces. Examples of these methods include coating or functionalizing with polyethylene glycol [12], poly(vinyl alcohol) [13], polyelectrolytes [14], and zwitterionic polymers [15]. However, superhydrophilic surfaces can be contaminated by hydrophilic (polar) pollutants, which could encompass both biological (microorganisms) and non-biological (soil) origins. To address this drawback, the development of superhydrophobic surfaces has been proposed. Superhydrophobicity is influenced by the structural aspects of the surface, particularly the low surface energy that arises from a hierarchical structure [16]. These patterns consist of regular nano-scale textures, such as hills, bumps, valleys, and pores found on the surfaces of plants like lotus leaves or rice leaves and on insect body parts like butterfly wings or diving beetle cuticles [17]. Air pockets are trapped between these protruding structures, reducing the solid–liquid interface area and enhancing the degree of hydrophobicity [18]. However, a limitation of superhydrophobic surfaces is their susceptibility to contamination by substances with low surface tension, which can easily penetrate and contaminate these surfaces. Therefore, the omniphobic property, which includes both hydrophobicity and oleophobicity simultaneously, is important. An omniphobic surface possesses an extremely low surface energy, dramatically reducing the real contact points and endowing a unique non-wetting characteristic, wherein the surface repels both polar and non-polar contaminants. This versatile nature renders omniphobic surfaces valuable in various applications such as anti-adhesion [19], anti-icing [20], microfluidic flow control [21], heat transfer control [22], and oil–water separation [23].

When oil is involved, achieving an omniphobic surface is challenging because oil, with a surface tension ranging from 20 to 50 mN·m^−1^, typically exhibits lower surface tension (72 mN·m^−1^) than water [24]. Previous studies on re-entrant structures [25] and double re-entrant structures [26] have provided guidelines for fabricating omniphobic surfaces. However, the complex processes involved in producing such nano-scale hierarchical structures are a drawback [27]. Examples of these processes include top-down methods such as lithography [28], etching [29], anodization [30], and laser processing [31], as well as bottom-up methods such as electrospinning [32], nanofiber self-assembly [33], sol-gel processing [34], and spray deposition [35]. While top-down methods like lithography are effective in creating omniphobic surface structures, the high equipment costs limit their industrial applicability. Similarly, bottom-up methods like electrospinning have limitations such as the dependency on the types of substrates, low production efficiency due to stringent manufacturing conditions, and lack of material durability. However, in the top-down method of dip-coating [36], which involves the deposition of nano-scale particles onto the surface, is widely used because of its simplicity. It facilitates the modification of surface properties to achieve different levels of wetting, regardless of the material or shape of the substrate [37].

In this study, the primary objective was to develop an omniphobic PU leather surface capable of repelling both polar and non-polar contaminants simultaneously. Nano-silica particles were functionalized with trichloro(1H,1H,2H,2H-perfluorooctyl)silane and applied as a coating onto the PU leather surface to create nano-scale structures with omniphobic characteristics. The surface exhibited high contact angles against a range of polar and non-polar substances, including water, n-hexadecane, bacterial suspensions, tannic acid, squalene, and mud. The modified surface was subjected to physical and chemical analysis by scanning electron microscopy (SEM), Fourier transform infrared (FTIR) spectroscopy, and tensiometry. To evaluate its effectiveness in preventing biofouling and facilitating self-cleaning against infectious microorganisms, the surfaces were inoculated and then subjected to culture for testing purposes. The results showed significant promise across various fields demanding improved hygiene standards. This included the resistance of the surface to mechanical wear, cold and heat treatments, and UV exposure.

## 2. Materials and Methods

### 2.1. Leather Samples and Chemicals

The polyurethane (PU) synthetic leather used for the substrate was purchased from BL Chemical (Gwangju-si, Republic of Korea) and cut into 10 × 10 × 1 mm dimensions for use. To sterilize the samples, 99.5% ethanol obtained from Samchun Chemicals (Seoul, Republic of Korea) was used for washing, followed by air-drying at room temperature (21 °C). Nano-silica (SiO_2_) particles, with an average diameter measuring 20 nm, were obtained from Sigma-Aldrich (St. Louis, MO, USA) for surface coating. Additionally, trichloro(1H,1H,2H,2H-perfluorooctyl)silane (TPFS), also sourced from Sigma-Aldrich, was utilized to fluorinate the surface of the nano-silica particles. n-Hexane, sourced from Samchun Chemicals, was utilized as a solvent for both the reaction and dispersion of the nano-silica particles.

### 2.2. Preparation of FNPs Suspension

For the preparation of fluorine-functionalized nano-silica particles (FNPs) solution, 300 mg of nano-silica particles and 15 mM of TPFS were reacted in 50 mL of n-hexane solvent. Subsequently, the mixture was sonicated for 20 min using the WUC-D03H ultrasonic equipment (Daihan Scientific, Wonju-si, Republic of Korea) to ensure uniform dispersion. After dispersion, the solution was allowed to stand for 1 h at room temperature to ensure sufficient chemical silanization of the nano-silica particles.

### 2.3. FNPs Deposition by the Dip-Coating Process

The dip-coating method was employed, which includes precise and controlled immersion and withdrawal, to deposit FNPs onto the PU leather surface. The process involved immersing the previously prepared coating solution for 30 s and drying for 1 min, repeated five times. Immersion and withdrawal speed during the dip-coating process were set at a speed of 1 mm/s to ensure uniform coating formation. Leather samples coated with FNPs were dried for 24 h at room temperature, resulting in a coating thickness of approximately 1.6 μm (please see Figure S1 in the Supplementary Materials).

### 2.4. Surface Characterization

To examine the morphology of the coated PU leather surface and confirm the uniformity of the coating, an S-5200 field emission scanning electron microscope (FE-SEM, Hitachi, Tokyo, Japan) was utilized. The SEM was operated at an acceleration voltage of 15 kV and emission current of 10 μA. To reduce surface charge accumulation, causing distortion in the captured SEM images, the sample surface was coated with a 15 nm thick layer of platinum (Pt).

The chemical state of material surfaces was analyzed using Fourier transform infrared (FTIR) spectroscopy. The data, obtained from the Nicolet iS10 FTIR spectrometer (Thermo Fisher Scientific, Waltham, MA, USA), were processed and analyzed using the included OMNIC Specta version 9.9.509 software.

The surface energy in terms of hydrophobicity and oleophobicity of the PU leather surfaces was determined by measuring the static contact angle using a tensiometer (Phoenix 300 Touch, SEO, Siheung-si, Republic of Korea). Sterile deionized (DI) water (with a resistivity of 18.2 MΩ·cm) and n-hexadecane (Sigma-Aldrich) were utilized for water contact angle measurements. Contact angles were obtained by dropping 5 μL of DI water and n-hexadecane onto the leather surface at least five times each, using the sessile drop method. The acquired contact angles were analyzed using the ImageJ version 1.53v software (National Institutes of Health, Bethesda, MD, USA) to calculate an average value. All contact angle measurements were conducted at room temperature.

The Wenzel roughness factor, which represents the surface area ratio, was calculated using the LEXT OLS4100 confocal laser scanning microscope (CLSM, Olympus, Tokyo, Japan).

### 2.5. Bacteria Cultivation

The two bacterial strains used in the experiment, *Escherichia coli* O157:H7 (Gram-negative, ATCC 25922) and *Staphylococcus epidermidis* (Gram-positive, ATCC 12228), were obtained from the Korean Culture Center of Microorganisms (KCCM, Seoul, Republic of Korea). Bacteria cultured on tryptic soy agar (TSA, BD, Franklin Lakes, NJ, USA) were inoculated by transferring a 10 μL loopful into 9 mL of tryptic soy broth (TSB, BD). After an initial incubation period at 37 °C for 24 h, the process was repeated once more using a fresh batch of TSB for another 24 h incubation period. The final concentration of the cultured bacteria obtained was found to be between 8.6 and 9.2 log CFU/mL.

### 2.6. Inoculation of Leather Surfaces and Bacterial Anti-Adhesion Assay

To compare the anti-biofouling properties between bare leather samples and leather samples coated with FNPs, the samples were immersed for 4 h in 5 mL of bacterial suspension. The degree of bacterial adhesion on the surfaces of bare leather samples and FNPs-coated leather samples were evaluated using the pour plate method. For the evaluation, the samples immersed in the bacterial suspension were placed in 9 mL of 0.1% (*w*/*v*) buffered peptone water and vortexed for 1 min to detach the bacteria adhering to the surface. The bacteria contained in the diluted peptone water were cultured with TSA under aerobic conditions at 37 °C for 24 h. The bacterial distribution among leather samples denotes the quantity of bacteria adhering to the surfaces.

Additionally, SEM enabled the observation of the bacteria adhered to the PU leather surface. The samples were immersed in the bacterial suspension for 4 h and then removed for examination. For SEM imaging, the bacteria were fixed using ethanol and coated with a thin layer of Pt, with a 15 nm thickness, to enhance the surface conductivity of the samples, thereby improving the clarity of images.

### 2.7. Self-Cleaning Efficiency of Leather Surfaces

To confirm the self-cleaning characteristics of the FNPs-coated sample surface, a measurement was carried out by applying contaminants to the substrate surface and subsequently dropping a specific amount (0.5 mL) of DI water and squalene. The sliding angle of the substrate was 1°, and tannic acid was used as the polar contaminant, while squalene was used as the non-polar contaminant, with Oil Red O (Sigma-Aldrich) dye added.

### 2.8. Thermal Stability Test of Leather Surfaces

To analyze the thermal stability of FNPs coating, bare leather and FNPs-coated leather were cooled or heated, and the contact angle was measured. To confirm stability, heat was applied from −10 °C to 80 °C at intervals of five times for 1 h, and the surface temperature was confirmed using infrared thermometer.

### 2.9. Mechanical Durability Test of Leather Surfaces

In order to verify the durability of the FNPs coating, a tape peel test was conducted. The tape peel test involved attaching an adhesive sheet to the bottom of a 500 g counterweight and repeatedly peeling it off at a speed of 3 mm/s for 50 cycles. Subsequently, to assess the maintenance of the coating, mud with relatively high viscosity was sprinkled on the slightly tilted (θ = 3°) sample surface to confirm if the self-cleaning properties were retained.

### 2.10. Photostability Test of Leather Surfaces

The resistance of the coating to photodegradation was accomplished by subjecting it to ultraviolet (UV) irradiation. The coated leather was exposed to UV with a wavelength (λ) of 365 nm for 1 h at room temperature. UV exposure was conducted through multiple passes at a conveyor belt speed of 10 m/min under ambient air conditions, utilizing a 120 W/cm mercury (Hg) lamp positioned at a distance of 30 cm from the belt, resulting in an energy density of 210 mJ/cm^2^ per pass.

### 2.11. Statistical Analysis for Analyzing Bacterial Enumeration

The results of the previous bacteria experiment were converted to log CFU/mL for the bacterial population, and the mean and standard deviation were calculated. A one-way analysis of variance (ANOVA) with Tukey’s post hoc test was conducted to determine if there were significant differences in the microbiological results between the bare surface and the coated surface, with a significance level of 0.05. All statistical analyses were carried out using the jamovi (version 1.1) software (jamovi, Sydney, Australia).

## 3. Results and Discussion

### 3.1. Characterization of FNPs-Coated Leather Surfaces

**Figure 1:**
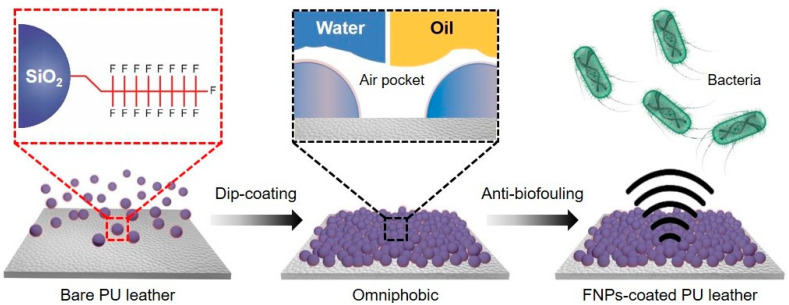
A schematic depiction of modifying the PU leather surface with FNPs to achieve omniphobic characteristics with anti-biofouling properties.

Using the dip-coating technique illustrated in Figure 1, FNPs were applied to a leather surface to create a nanostructured omniphobic surface that effectively prevented bacterial attachment. Previous studies have indicated a significant association between the nano-scale roughness of surfaces and the adhesion of bacteria [38]. Bacteria preferably adhere to curved surfaces with larger contact areas. Therefore, when the surface roughness exceeds the average size of bacteria (typically 0.5–2.0 μm in length or diameter), bacterial colonization is promoted by physical entrapment or sticking [39].

**Figure 2:**
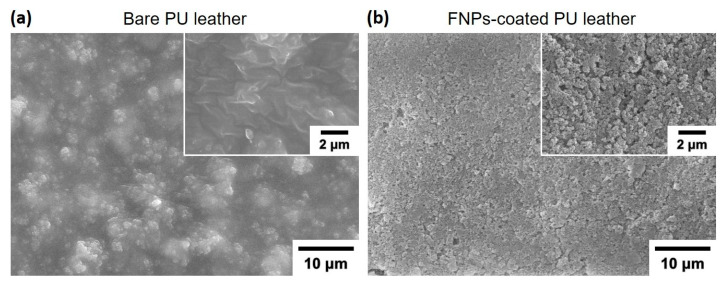
SEM images of (**a**) bare leather and (**b**) FNPs-coated leather surfaces from a top-view perspective.

SEM images (Figure 2) of the bare and FNPs-coated PU leather surfaces reveal the formation of porous nanostructures on the FNPs-coated surface, with air pockets between the particles. These air pockets create gaps at the interface, reducing adhesion and ensuring favorable conditions characterized by low surface energies [40]. These nano-scale structures play a crucial role in repelling liquids. When a liquid droplet is placed on a nanorough surface, it contacts the higher parts of the nanorough structure, while air trapped in the lower parts prevents the droplet from penetrating into the voids [41]. The air trapped in these nano-scale structures with a large volume creates resistance to both polar and non-polar contaminants, preventing their infiltration. However, the air pockets alone are not sufficient for lowering the surface energy to prevent adhesion against contaminants.

**Figure 3:**
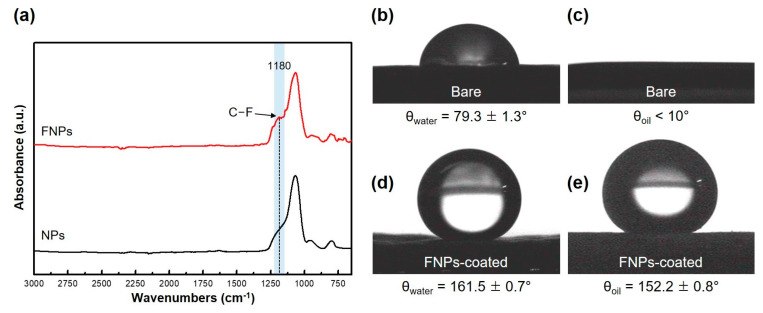
(**a**) The FTIR spectra for both FNPs and pristine nano-silica particles (NPs), indicating the chemical composition of the materials. (**b**–**e**) Contact angle images of water and oil (n-hexadecane) on the bare and FNPs-coated leather surfaces.

To further reduce the surface energy, fluorinated silane was functionalized on nano-silica particles with a strong binding affinity [42]. The trifluoromethyl functional group (–CF_3_) of the FNPs is more effective in reducing the surface energy than substances with hydrogen atoms or relatively low fluorine distribution. In typical cases, the surface energy decreases in the order of –CH_2_ (36 mJ/m^2^) > –CH_3_ (30 mJ/m^2^) > –CF_2_ (23 mJ/m^2^) > –CF_3_ (15 mJ/m^2^) [43]. Bacteria can adhere to surfaces with both hydrophilic and hydrophobic characteristics; however, they tend to adhere more readily to hydrophilic surfaces [44]. In this study, the molecular interactions between bacteria and the surface were reduced by coating the leather surface with fluorine-based compounds. FTIR spectroscopy (Figure 3a) was used to confirm the chemical bonding between fluorinated silane and nano-silica particles. When fluorinated silane reacted with nano-silica particles, the FTIR spectrum showed a C–F peak at 1180 cm^−1^, which was absent in the spectrum of the untreated nano-silica particles. This confirmed the successful functionalization of the fluorinated silane on the surface of the nano-silica particles [45]. These results confirm the successful functionalization of fluorinated silane on the surface of nano-silica particles. Through functionalization, chemical covalent bonds were formed between the accumulating particles and reagent, allowing the formation of mechanically durable and stable surfaces. Moreover, the method did not require additional surface treatments to enhance the adhesion between the substrate and the coated material, as the result of the radical polymerization of FNPs, fluoropolymer, served as an energetic binder [46,47]. Hence, through chemical functionalization, it is possible to fabricate an omniphobic surface with low surface energy and high durability.

### 3.2. Wettability of Omniphobic FNPs-Coated Leather Surfaces

The process of removing contamination from leather surface is complex, and such contamination can spread further as secondary contamination. In this regard, omniphobic surfaces can prevent contamination in advance and also prevent bacterial adhesion, rendering them beneficial for various industries [48]. To verify the successful modification of the surface topography and chemistry to achieve low surface energies, water and oil (i.e., n-hexadecane, a component of essential oil) were dropped on the bare and FNPs-coated leather surfaces to compare their wetting characteristics. Figure 3b–e shows the contact angles of water and oil droplets. On the bare leather surface, the water contact angle (WCA; θ_water_) and oil contact angle (OCA; θ_oil_) were measured to be 79.3 ± 1.3° and < 10°, respectively, confirming the vulnerability of the surface to contamination by water and oil. The WCA and OCA of the FNPs-coated leather surface increased significantly to 161.5 ± 0.7° and 152.2 ± 0.8°, respectively, confirming the omniphobic wetting characteristics. The wetting characteristics of a liquid on a nanostructured surface can be elucidated using the Wenzel state model and Cassie–Baxter state model theories [49].

(1) $$\cos\theta_{w}=R_{f}\cos\theta_{\gamma }$$

The Wenzel state model assumes that water droplets completely infiltrate the surface and expresses the surface roughness as the ratio of the projected area to the actual area of the surface in contact with the water droplet. In Equation (1), *θ_w_* and *θ_γ_* represent the expected contact angles in the Wenzel state model and Young’s model, respectively [50]. *R_f_* represents the roughness factor in the Wenzel state model when in contact with the liquid, and a value of 1.27 was obtained using CLSM. The Cassie–Baxter state model assumes that water droplets do not infiltrate the surface but are in contact with the air between the protruding parts [51]. The contact angle (*θ_c_*) of the water droplet on the surface is given as follows:

(2) $$\cos\theta_{c}=(f_{1}+1)\cos\theta_{\gamma }-1$$

Here, *f*_1_ represents the liquid–solid contact area ratio [52]. According to this theory, the WCA in the Wenzel state model was calculated to be as low as 87.7°, which was lower than the measured actual contact angle of 161.5°. This indicated that the water droplets in the nanostructures of the surface were not absorbed between the protrusions, trapping air beneath the water or oil. Therefore, the coated surface obeyed the Cassie–Baxter model [53]. Additionally, the experimental results suggested that the combination of the air pockets and the exposure of functional groups on the surface contributed to the formation of the omniphobic surface.

### 3.3. Anti-Biofouling Properties of FNPs-Coated Leather Surfaces

**Figure 4:**
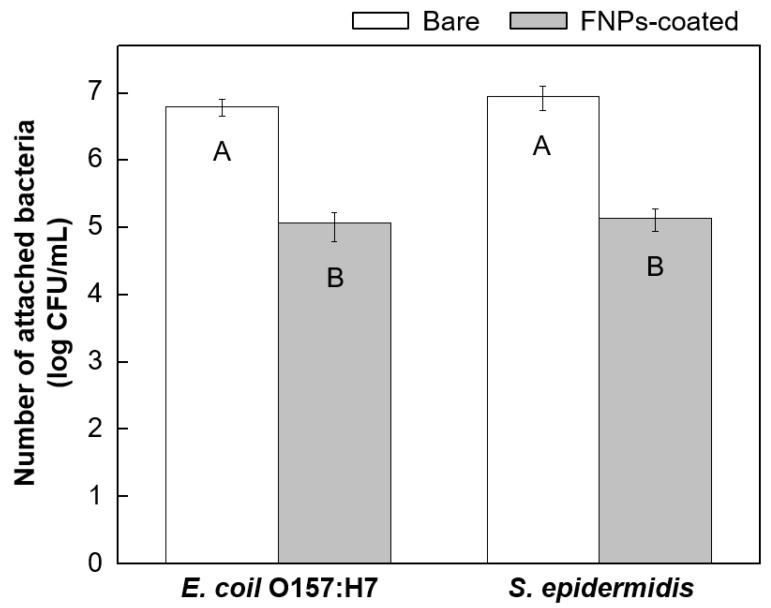
The bacterial attachment on both bare and FNPs-coated leather surfaces for *E. coli* O157:H7 and *S. epidermidis* was quantified after a 4 h immersion in bacterial suspension, using the pour plate method. Statistical significance is indicated by different letters, with *p* < 0.05.

After analyzing the surface characteristics of the coated PU leather, bacterial adhesion properties of the bare and coated surfaces were compared (Figure 4). The graph represents the plate count measurements of bacteria adhered to the bare and coated leather surfaces. As shown in the left graph, the adhesion of *E. coli* O157:H7 on the bare leather surface was 6.8 ± 0.2 log CFU/mL, while that on the coated leather surface significantly decreased to 5.1 ± 0.3 log CFU/mL. This bacterial adhesion was more preferred on the bare leather surface compared to that on the coated leather surface, with the bacterial adhesion decreasing by more than 98.2% numerically on the latter. Upon comparing the means using ANOVA, the bare and FNPs-coated leather surfaces showed statistically significant differences in the adhesion of *E. coli* O157:H7 (*p* < 0.05). A similar trend was observed using *S. epidermidis*. The right graph represents the plate count measurements of *S. epidermidis* adhered to the bare and FNPs-coated leather surfaces. The number of bacteria adhered to the bare and coated leather surfaces was 7.0 ± 0.1 log CFU/mL and 5.2 ± 0.3 log CFU/mL, respectively, indicating a significant reduction in the adhesion by more than 98.5%. ANOVA revealed significant differences in the adhesion levels of *S. epidermidis* on the two surfaces (*p* < 0.05). The pour plate method experiment revealed that compared to bare leather, the number of bacteria adhered to the FNPs-coated leather decreased by more than 1.7 log CFU/mL. Furthermore, two-way ANOVA confirmed that there were no statistically significant differences in the adhesion behavior of the two bacterial species on the leather surface (*p* < 0.05).

**Figure 5:**
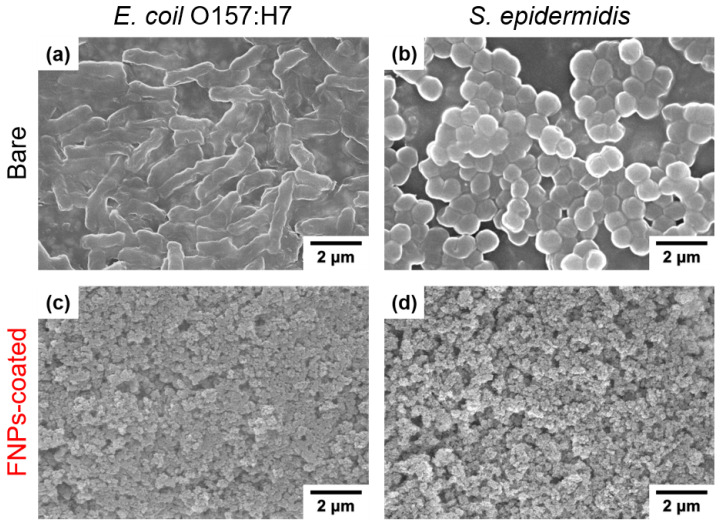
SEM images of bacterial attachment on bare and FNPs-coated leather surfaces for (**a**,**c**) *E. coli* O157:H7 and (**b**,**d**) *S. epidermidis* following a 4 h exposure to bacteria.

To visually confirm the degree of bacterial adhesion, we employed SEM to directly quantify the adhered bacteria on each leather surface. Figure 5a,c shows the SEM images of the leather surface after inoculating with an *E. coli* O157:H7 suspension for 4 h. The mean bacterial densities on the bare and FNPs-coated leather surfaces exposed to the bacterial suspension for 4 h were 5.8 ± 0.1 log cell/mm^2^ and 3.6 ± 0.2 log cell/mm^2^, respectively. These results indicated a reduction in bacterial contamination by over 99.4%. Additionally, the mean bacterial densities of the adhered *S. epidermidis* on bare and coated leather samples after immersion in *S. epidermidis* suspension for 4 h were determined to be 5.9 ± 0.2 log cell/mm^2^ and 3.8 ± 0.0 log cell/mm^2^, respectively, from the SEM images (Figure 5b,d), confirming a reduction in bacterial adhesion by over 99.2%.

The plate count results and SEM images demonstrated that the FNPs coating on the leather significantly reduced bacterial adhesion. This trend was consistent with the shift in the wetting behavior from the Wenzel state model to the Cassie–Baxter state model upon FNPs coating. The transition suggested the formation of air pockets between the leather surface and liquid, which decreased the contact area and initial adhesion force between the bacterial suspension and leather surface, thereby indicating a change in the surface hydrophobicity [54,55]. Most bacterial cell walls possess hydrophilic groups on their surfaces. Consequently, the adhering of bacteria to omniphobic surfaces is difficult because of the repulsion between the bacteria and surfaces, specifically in biointerfaces [56].

### 3.4. Self-Cleaning Properties of FNPs-Coated Leather Surfaces

**Figure 6:**
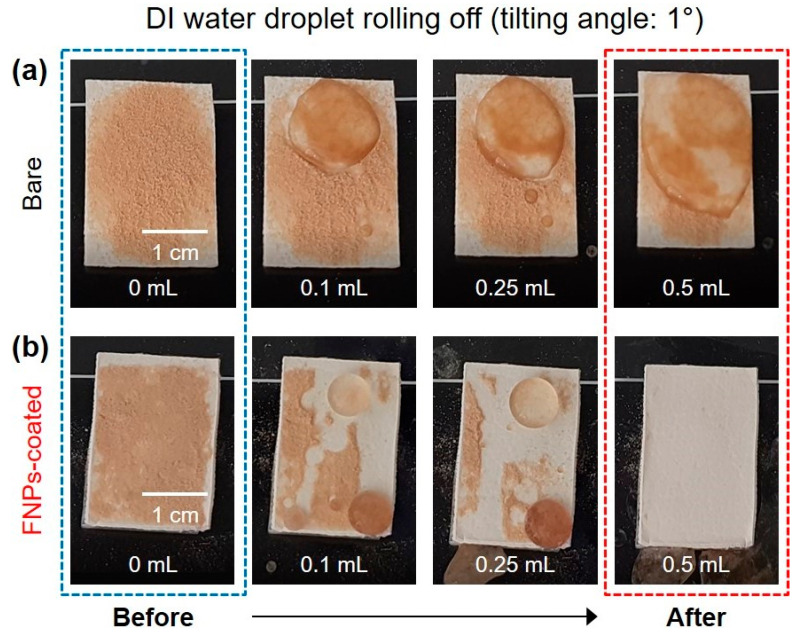
Self-cleaning properties were evaluated by applying water onto both (**a**) bare and (**b**) FNPs-coated leather surfaces with tannic acid present.

A self-cleaning surface is any surface that can easily remove dirt, grime, debris, and other contaminants. For leather surfaces to be effectively applied as materials that can remove external sources of contamination, it is important to possess the self-cleaning property, by the virtue of which both polar and non-polar contaminants are repelled from the surface [57]. Fine particles like dust typically exhibit hydrophilic surface properties. Thus, when a liquid is dropped on an inclined omniphobic surface, the liquid collects the dust and easily rolls off, facilitating dust removal. In this study, tannic acid, chosen as a representative substance derived from plants, served as the contaminant. This compound was selected based on the relatively strong binding between tannic acid and material surfaces, attributable to the abundant hydroxyl groups present in its chemical structure [58]. The objective was to assess the self-cleaning ability of the FNPs-coated leather surface. For this, 0.5 mL of deionized water was dropped with a sliding angle of 1° onto the FNPs-coated leather surface that was sprayed with tannic acid powder (Figure 6). Due to the nanostructure of the surface and the low surface energy of the fluorinated functional groups, water droplets maintained a spherical shape and easily rolled off the omniphobic surface [59]. Furthermore, the tannic acid powder was absorbed by the water droplets and removed from the coated surface along with the water droplets. Therefore, it was confirmed that the FNPs-coated leather surface exhibited omniphobicity-based anti-biofouling properties as well as self-cleaning properties.

**Figure 7:**
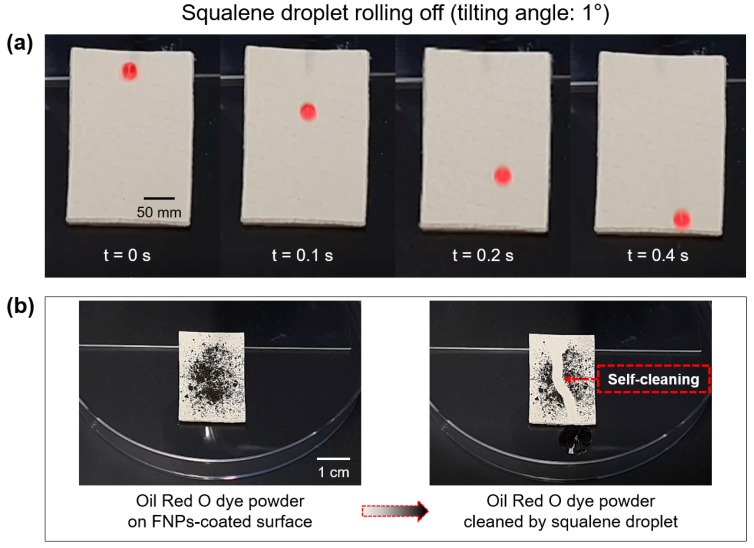
Self-cleaning capabilities were assessed by applying squalene onto the leather surface coated with FNPs. (**a**) Squalene dyed with Oil Red O was observed to roll off from the coated surface. (**b**) Oil Red O dye powder was placed on the coated surface, demonstrating that contaminants were easily cleaned by squalene droplets.

Experiments were also conducted to confirm the self-cleaning properties with oil. Figure 7a demonstrates squalene, containing a small amount of Oil Red O dye powder, easily rolling off the surface at a sliding angle of 1°. To confirm the self-cleaning property, 0.5 mL of squalene was dropped after sprinkling 50 mg of Oil Red O dye powder—a hydrophobic contaminant—on the FNPs-coated leather surface (Figure 7b). Squalene is a hydrophobic substance that is widely used in fingerprint prevention tests and is similar to the oil released off the skin [60]. When squalene was dropped on the omniphobic surface, the liquid maintained its circular shape and was continuously removed, along with the surrounding hydrophobic contaminants. This confirmed that the FNPs-coated surface exhibited self-cleaning properties against both water- and oil-based contaminants.

### 3.5. Thermal Stability of Leather Surfaces

**Figure 8:**
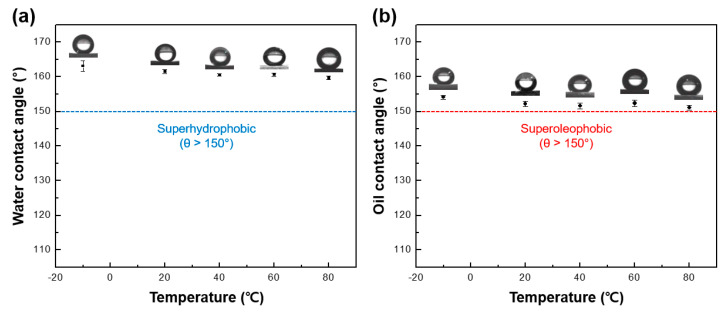
The (**a**) water contact angle and (**b**) oil contact angle of the FNPs-coated leather surfaces were measured at varying temperatures to assess the temperature-dependent behavior of surface wettability.

Coatings are often exposed to low or high temperatures, leading to their damage [61,62]. Leather, in particular, is frequently exposed to various temperature conditions. Thus, it is imperative for an omniphobic surface to possess thermal stability. Figure 8 displays the contact angles of the liquids following the exposure of the sample to temperatures ranging from −10 to 80 °C. The samples were exposed for 1 h at five different temperatures in this temperature range, and the contact angles of water and oil were measured. Angles exceeding 150°, corresponding to superhydrophobic or superoleophobic properties, were maintained at all temperatures, indicating that the omniphobic surface possessed excellent thermal stability.

### 3.6. Mechanical Durability and Photostability of Leather Surfaces

**Figure 9:**
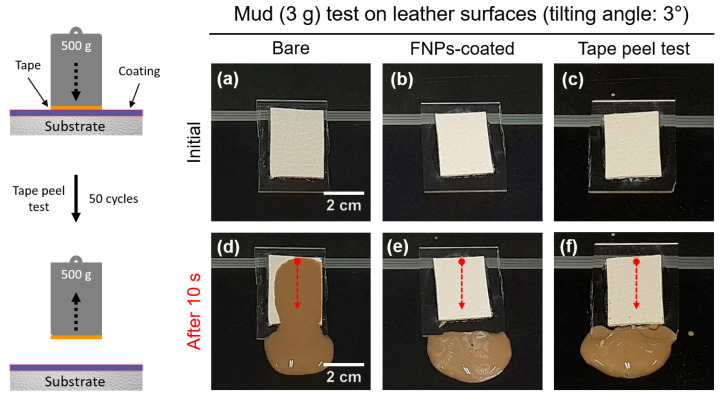
A schematic illustration of tape peel test and self-cleaning properties of leather surfaces against mud on the (**a**,**d**) initially bare, (**b**,**e**) initially FNPs-coated, and (**c**,**f**) FNPs-coated leather surfaces after 50 cycles of tape peeling. The red dot arrows indicate the direction of transport for the mud contaminants.

Ensuring highly durable omniphobic properties is crucial, as surface coatings are susceptible to damage from mechanical impacts. Adhesion testing is one such test that should be performed for determining the durability, as insufficient adhesion of the coating to the substrate can cause the coating to separate from the substrate. The tape peel test, being simple and practical, was used in this study to measure the mechanical durability [63]. During the durability test, the tape was repeatedly attached and detached to determine whether its self-cleaning capabilities were preserved when mud was applied to the surface. According to several studies, car seat cushions typically measure 410 mm in width and 570 mm in height. The weight applied to the seat was calculated to be 40 g per unit area, considering the average weight of an adult and the contact area [64]. To assess the self-cleaning properties, the FNPs-coated leather underwent 50 cycles of attachment and detachment with a weight of 500 g, which is significantly higher than the calculated load of 40 g. Figure 9 shows a photograph of the mud test; relatively strong adhesion of mud compared to the case of low viscosity contaminants was realized. This demonstrated that the coated leather maintained its self-cleaning properties, as the mud easily flowed off even at a small sliding angle of 3°, while the bare leather was contaminated with mud. This confirmed that the FNPs coating provided sufficient mechanical durability.

One of the prominent surface treatment methods for leather in its production and processing is to coat using representative photocurable polymer materials [65]. Therefore, to verify the stability under light exposure, UV light generated from a Hg lamp was irradiated on the samples at a conveyer belt speed of 10 m/min and output of 120 W/cm. The contact angles of the FNPs-coated leather surface before and after the UV treatment (Table 1) confirmed that the omniphobic properties were maintained.

## 4. Conclusions

This study focused on preventing biological and non-biological contamination by imparting water and oil repellency to the PU leather surfaces, commonly used in daily life, by simply coating them with FNPs. By adjusting the surface chemistry and nano-scale morphology, we achieved anti-wetting properties with WCA and OCA higher than 150°, indicating omniphobic characteristics. A low surface energy fluorosilane layer, formed via functionalization reactions, repelled liquids, while the nano-silica particles on the surfaces created nanostructures that minimized contact points. Notably, we simultaneously achieved several desirable properties: (1) anti-biofouling property, (2) self-cleaning property, (3) low/high thermal reliability, (4) mechanical stability, and (5) photostability. The anti-biofouling property was verified using the dip-inoculation method, revealing that bacterial fouling of the FNPs-coated surface decreased by over 98.2% compared to that of the bare surface. We believe that the coating developed in this study will have wide-ranging applicability, extending beyond leather to various other materials. This versatile application is expected to generate considerable interest across a range of protection, cleanliness, and hygiene-related applications.

## Figures and Tables

**Table 1:** Contact angle measurements were conducted on both bare and FNPs-coated leather surfaces before and after exposure to UV.

Sample	Liquid	UV Exposure Time	CA_water_ (θ, before UV)	CA_oil_ (θ, after UV)
FNPs-coated leather	Water	1 h	160.2 ± 0.3°	158.3 ± 0.6°
FNPs-coated leather	Oil	1 h	152.1 ± 0.5°	151.7 ± 0.7°

CA (θ): contact angle of water and oil determined by the sessile droplet method, before and after exposure to UV for 1 h.

## Data Availability

Data are contained within the article and Supplementary Materials.

## Supplementary Materials

The following supporting information can be downloaded at https://www.mdpi.com/article/10.3390/polym16141983/s1, Figure S1. The surface morphology and thickness of the coatings were observed using a confocal laser scanning microscope. A difference of approximately 1.6 µm in height (h) was observed between the bare PU leather and the FNPs-coated PU leather surfaces, indicating the thickness of the coating.
