# Supplementary material for: Durable, Photostable Omniphobic Synthetic Leather Surfaces with Anti-Biofouling Properties for Hygienic Applications
Source: Polymers (Basel). 2024 Jul 11;16(14):1983. doi: 10.3390/polym16141983 (PMC11281141; doi:10.3390/polym16141983)
Supplement: Supplementary file 1 [file polymers-16-01983-s001.zip › polymers-3052124-supplementary.pdf]

Durable, Photostable Omniphobic Synthetic Leather Surfaces with Anti-Biofouling Properties for Hygienic Applications

Hanna Lee and Jun Kyun Oh*

Department of Polymer Science and Engineering, Dankook University, Yongin-si 16890, Republic of Korea

*Correspondence and requests for materials should be addressed to J.K. Oh (email: junkyunoh@dankook.ac.kr).

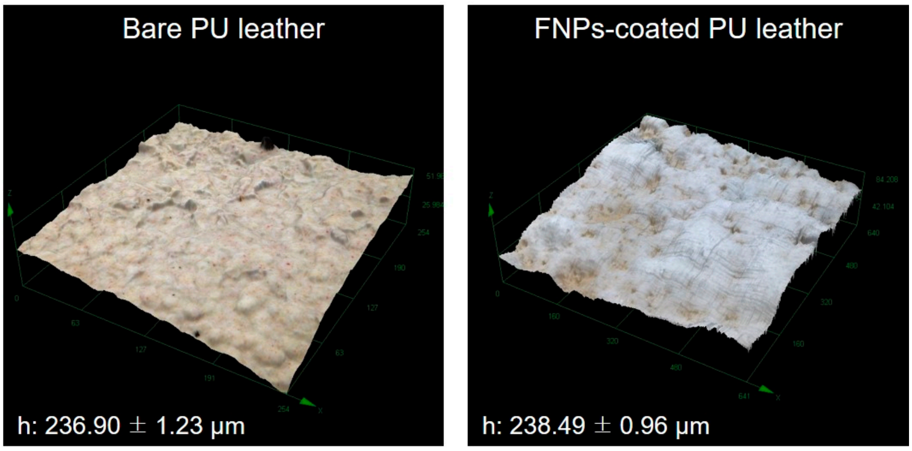

Figure S1. The surface morphology and thickness of the coatings were observed using a confocal laser scanning microscope (CLSM, Lext OLS4100, Olympus, Tokyo, Japan). A difference of approximately 1.6 μm in height (h) was observed between the bare PU leather and the FNPs-coated PU leather surfaces, indicating the thickness of the coating**.**
